# Reimagining the 5Ms: A Novel Application of the 5Ms Framework to Teach Residents How to Manage Medical Complexity Across the Lifespan

**DOI:** 10.7759/cureus.96656

**Published:** 2025-11-12

**Authors:** Emily N Bufkin, Kylie Cullinan, Jessica Voit

**Affiliations:** 1 Department of Internal Medicine, University of Texas Southwestern Medical Center, Dallas, USA

**Keywords:** 5ms, chronic diseases of childhood origin, complexity of care, curriculum framework, disability education, geriatrics and internal medicine, intellectual disability (id), medical resident education, med-peds, neurodiversity

## Abstract

Introduction

With advances in medicine, physicians care for an increasingly complex population. To prepare resident physicians to practice independently, they must receive formal instruction on managing medically complex patients. While the 5Ms framework of age-friendly care was developed to address the comprehensive and complicated needs of older adults, it can be adapted to optimize care for complex patients of any age. The 5Ms framework includes five domains: Mobility, Medications, Mentation, Multicomplexity, and Matters Most.

Methods

The authors developed a workshop to teach internal medicine residents a novel approach to organizing the care of complex patients across the lifespan. Case-based learning was combined with literature review and clinical application of learned topics. Learners were encouraged to participate in anonymous pre- and post-workshop surveys, where they reported their comfort level with the 5Ms framework and their confidence in outpatient clinical scenarios of adult patients with varying medical complexity.

Results

Paired survey responses were analyzed (n=76). Before the workshop, approximately 50% of learners were aware of the 5Ms framework, and only two residents felt "very comfortable" or "extremely comfortable" with it. After the workshop, comfort level with the 5Ms framework improved, with 51% of respondents reporting their comfort level as "very comfortable" or "extremely comfortable." Residents also reported increased confidence in their ability to manage every case scenario. Approximately 97% of residents said they would use the 5Ms framework post-workshop.

Discussion

This innovative workshop demonstrated that the 5Ms framework can be applied to complex patients of all ages. It offers a useful tool for educating residents on how to organize their care of medically complex patients.

## Introduction

Teaching residents how to efficiently and effectively manage medical complexity is crucial to their education [1]. Due to advancements in medical care and improved survivability of many conditions, internists care for an increasingly medically complex population [2]. The field of geriatric medicine specializes in managing frail and complex older adults, and studies show that comprehensive geriatric assessments improve outcomes [3].  

The John A. Hartford Foundation and the Institute for Healthcare Improvement developed the 4Ms framework in 2017, an initiative that has been widely disseminated and implemented in geriatric settings to address the comprehensive and complicated needs of older adults. This framework was designed to facilitate the management of complexity by organizing patient needs into four domains: Mobility, Medications, Mentation, and Matters Most [4]. The 4Ms framework was expanded to the 5Ms framework by incorporating the domain of Multicomplexity, which highlights the importance of considering multimorbidity and complex biopsychosocial situations in the care of older adults [5, 6]. The 5Ms framework has been deemed of high value, and the most recent medical student geriatric competencies were organized by the 5Ms [7]. At the graduate medical education level, the 5Ms framework has been used as a tool for teaching Accreditation Council for Graduate Medical Education (ACGME) geriatric competencies [8] to residents in primary care [9]. It has also been incorporated into interprofessional geriatric educational initiatives [10]. Its application has been expanded beyond general medicine as other specialties that frequently manage medically complex patients, such as rheumatology and cardiology, have begun encouraging utilization of the 5Ms framework for the care of older adults [11, 12]. While the utility of this point-of-care framework has been well described in older adults, there is no current literature regarding the application of the 5Ms framework beyond the geriatric population. However, it can be readily adapted beyond geriatric medicine to care for medically complex patients of any age. The 5Ms framework may be an especially important tool in structuring visits for young adults with chronic childhood-onset disease (CCOD) after they transfer to the adult model of care, as general internists have traditionally experienced high discomfort in caring for this vulnerable patient population [13-15]. 

The authors presented a workshop at the Society of General Internal Medicine Annual Meeting on May 16, 2024. The workshop introduced the traditional 5Ms of age-friendly care and expanded its application to a broader range of complex patients, regardless of age. As the workshop was well-received per the post-workshop survey, it was adapted for internal medicine (IM) residents at our institution. This study aimed to teach resident physicians this novel application of the 5Ms framework to help care for medically complex patients across the lifespan and improve their confidence in complex clinical cases. 

## Materials and methods

Our university's Internal Medicine residency program dedicates time to ambulatory education sessions. The 5Ms Beyond Geriatrics workshop was developed by a group of three attending physicians (one geriatrician and two medicine-pediatrics primary care physicians). Residents attended one of the five 90-minute-long workshops with their ambulatory cohort from October 14 to November 11, 2024. The number of attendees per session averaged 15 to 20 resident physicians. 

**Figure 1:**
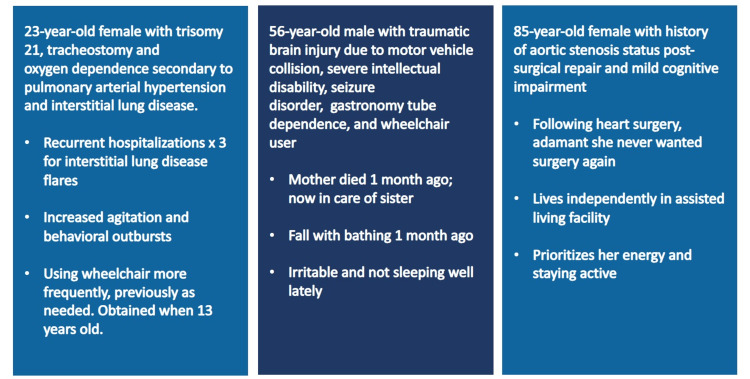
Case-based learning

The workshop's development was grounded in the constructivist experiential learning cycle to promote learner engagement [16]. Learners were expected to develop new knowledge of the 5Ms framework, beginning with reflection on prior clinical experience. Then, using a combination of case-based learning (Figure 1), a didactic review of literature, and a participatory discussion of clinical application, the workshop taught residents how to practically apply the 5Ms framework to organize the care of medically complex patients regardless of age. 

For the Mobility section, we reviewed the geriatric approach to a head-to-toe functional assessment, along with fall prevention strategies. This section was further expanded to include practical tips for caring for individuals who use wheelchairs, emphasizing the importance of collaborating with seating specialists to ensure an optimal fit and function. We also reviewed Centers for Medicare & Medicaid Services (CMS) guidelines regarding wheelchair prescription, coverage, and replacement. The Mentation section outlined dementia, mild cognitive impairment, and normal changes of aging, as well as differentiating delirium from dementia. We defined intellectual and developmental disability (IDD) and described the categories of mild, moderate, severe, and profound in relation to adaptive functioning and communication abilities. We also reviewed the diagnostic criteria for autism spectrum disorder, emphasizing the variability of symptoms in adults and outlining practical strategies for fostering more accessible and accommodating healthcare environments for neurodiverse individuals. In the Medications portion, we discussed deprescribing strategies to combat polypharmacy. We included a systematic approach to chronic constipation in medically complex patients, as well as contraception/menstrual suppression for vulnerable reproductive-capable women. The Multicomplexity section of the workshop focused on adapting guideline-based screening recommendations for complex patient populations through e-prognosis tools, the Geriatric Choosing Wisely campaign, and an established toolkit for IDD. In the Matters Most segment, we explored best practices for assessing perceived quality of life and defining goals of care in collaboration with patients and their support networks. We also discussed how to adapt these conversations to various support networks, including parents of aging young adults with CCOD, significant others or adult siblings, and adult children of an aging parent. 

**Figure 2:**
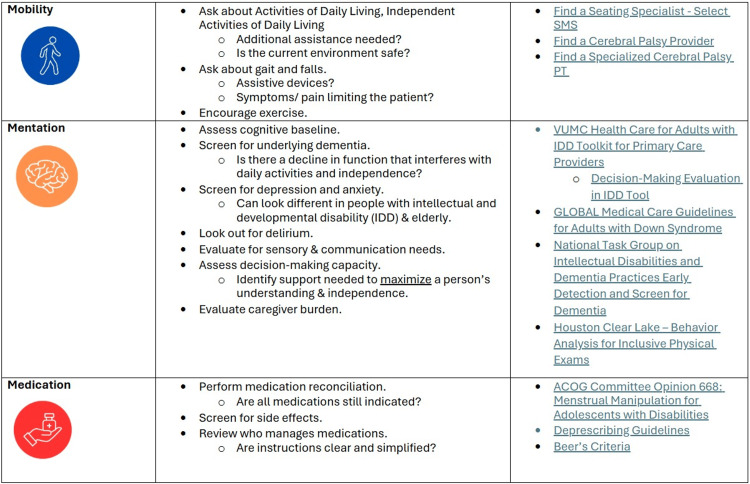
Resource guide (1) SMS - seating and mobility specialist; PT - physical therapy; IDD - intellectual and developmental disability; VUMC - The Vanderbilt University Medical Center; ACOG - American College of Obstetricians and Gynecologists

**Figure 3:**
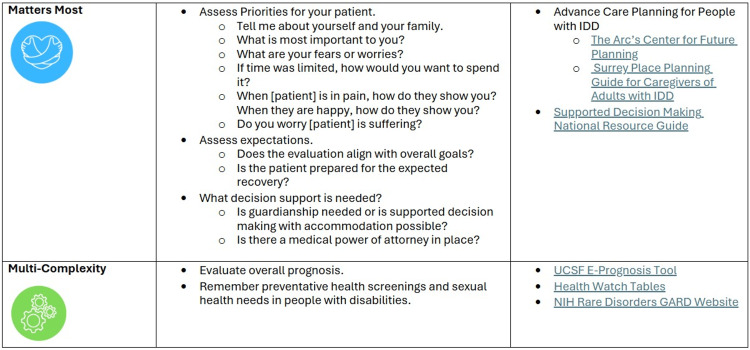
Resource guide (2) IDD - intellectual and developmental disability; UCSF - University of California San Francisco; GARD - Genetic and Rare Diseases Information Center

At the end of each section, we returned to the cases to allow learners to critically apply the material to case-study patients. To aid residents' future application of the framework, they received a resource guide containing online resources and a summary of the framework, including key aspects for each "M" (Figure 2, Figure 3).

Comfort with the 5Ms framework and resident confidence in managing outpatient complexity across the age spectrum were measured using an anonymized Microsoft Forms survey with pre- and post-intervention surveys. In the preintervention survey, learners created a unique identifier that allowed for the pairing of pre- and post-surveys. Respondents identified if they had awareness of the 5Ms Framework (yes/no) pre-intervention. Using branch point logic, those with pre-intervention awareness of the 5Ms Framework were asked to rate their comfort level with its application, with scores ranging from 1 (not at all comfortable) to 5 (extremely comfortable). All respondents were asked to rate their baseline comfort level when managing patients with varying complexity across the lifespan, with scores ranging from 1 (not at all comfortable) to 5 (extremely comfortable). After completing the workshop, learners were encouraged to complete an anonymous post-intervention survey to reassess their comfort level with the 5Ms framework and with example outpatient clinical scenarios. The Wilcoxon Signed-Rank test was used to assess statistical significance. This project was reviewed by the Institutional Review Board and determined to be non-regulated research. 

## Results

Ninety-one residents completed the pre-workshop survey, and 96 completed the post-workshop survey. After excluding respondents without both a pre- and post-survey, a total of 76 residents completed both the pre-intervention and post-intervention surveys. Fifty-three percent of the respondents were PGY-1 (n=40), 33% were PGY-2 (n=25), and 14% were PGY-3 (n=11). At the time of their workshop, approximately 41% (n=31) of the respondents had not yet received formal geriatric medical exposure during their residency. Before the workshop, 50% (n=38) were aware of the 5Ms framework. Of those learners, only two respondents rated their comfort level using the 5Ms as “very comfortable” or “extremely comfortable.” Only one respondent reported having used the 5Ms framework often. Approximately one-third of those respondents aware of the 5Ms had “never” used the framework (n=11), and approximately two-thirds had “sometimes” used the framework in their clinical practice (n = 26). When presented with outpatient case scenarios and asked to rate their confidence in management, residents reported feeling more confident with the less complex patients and with the more common diagnoses before the workshop. Notably, the scenario of the young adult with CCOD received the lowest preintervention confidence ratings, with only one respondent rating their comfort level as "very confident" or "extremely confident". 

**Figure 4:**
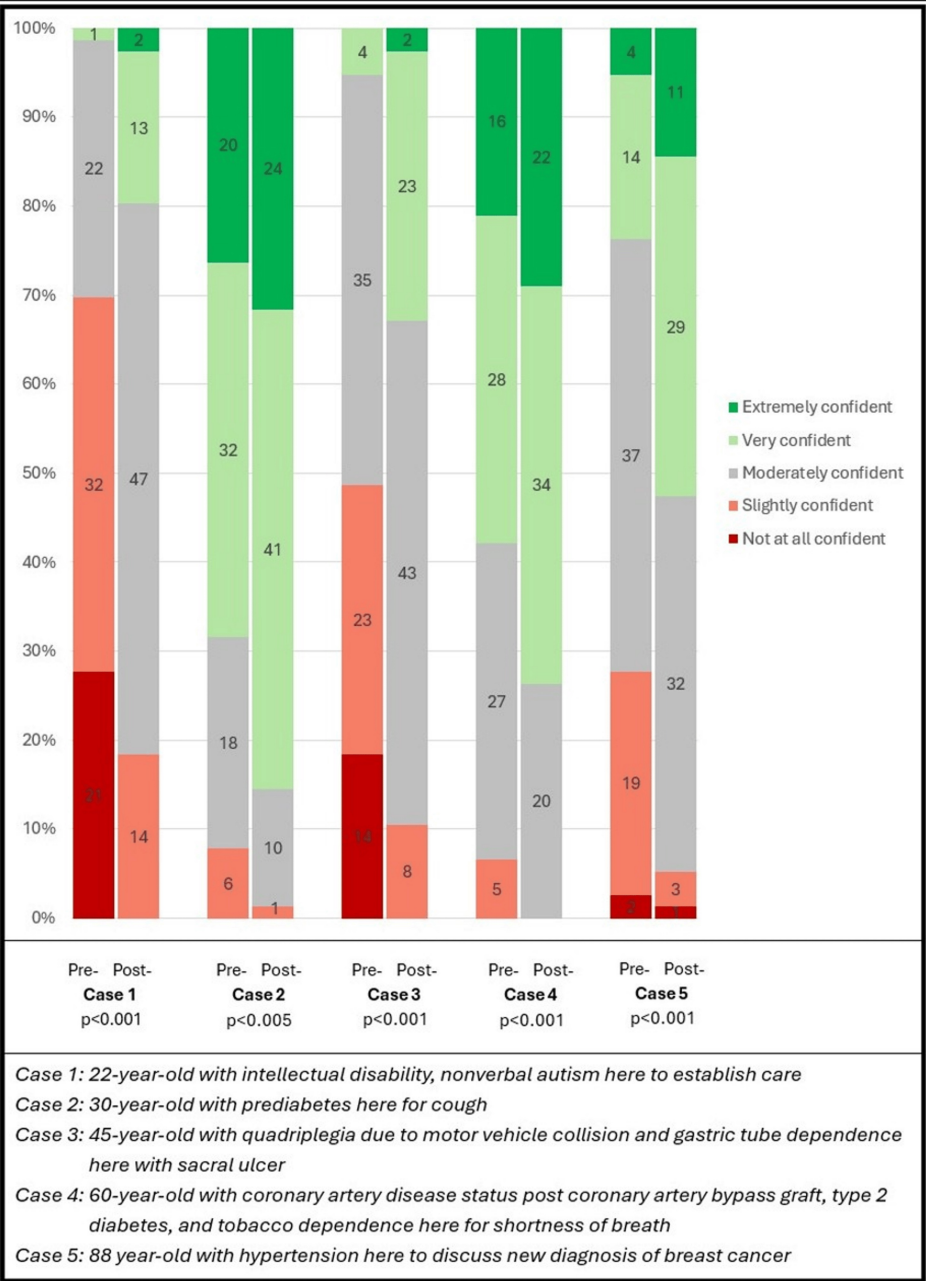
Resident Confidence with Cases

**Figure 5:**
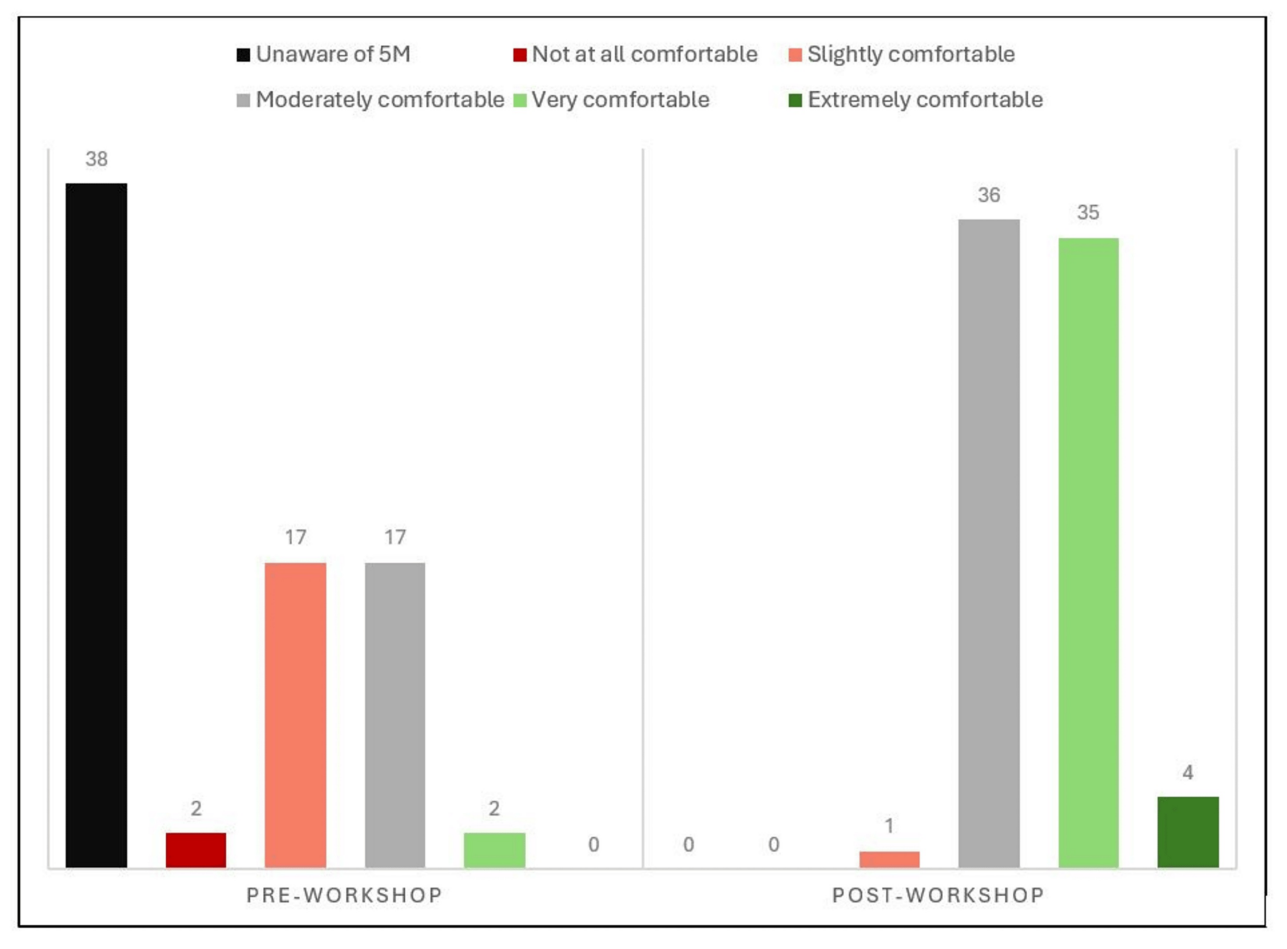
Resident Comfort Level with the 5Ms

After the workshop, learner self-reported confidence improved for all outpatient case scenarios (Figure 4). There was a significant difference in resident confidence for cases one, three, four, and five, with a p <0.001. For case two, there was a significant medium difference (p=0.005) between the pre- and post-intervention surveys. The comfort level using the 5Ms framework showed a significantly large improvement (p<0.001), with 51% of respondents (n=39) rating their comfort level as "very comfortable" or "extremely comfortable" (Figure 5). After the workshop, approximately 97% of the residents (n=74) stated that they would use the 5Ms framework in the future, with 59% (n=45) reporting that they would use it often.

In an optional free-text response to the survey prompt, "Please provide feedback on the workshop", residents expressed interest in applying this framework to their clinical practice as well as remarked on its usefulness in caring for complex patients of all ages. Demonstrative quotes include: *"Excellent workshop! I think [the] 5Ms are a great framework and I look forward to helping my patients with it."*;* "This was great to provide a framework for some tough situations!"*; and *"This was a great workshop. I learned many pearls about the 5Ms I plan to use to group problems together in a complicated patient."*

## Discussion

The 5Ms framework of age-friendly care is a tool intended to facilitate comprehensive yet time-efficient care of medically complex older patients. While traditionally geared toward geriatric medicine, the 5Ms can easily be applied to patients across the lifespan. To our knowledge, this is the first study application that expands the popular 5Ms framework to patients other than older adults. 

This pilot workshop suggests that this new application of the 5Ms framework is well-received and could improve residents' comfort in dealing with medically complex patients across the lifespan, as well as expand their knowledge of how to organize complex care. All cases showed significant improvements in resident confidence levels after the workshop. There were large confidence level changes in cases one, three, four, and five (p<0.001), underscoring the impact the 5Ms has on residents' confidence in organizing their outpatient approach to complex cases. This was particularly dramatic in the case of the patient with CCOD, where 53 trainees felt "not at all confident" or "slightly confident" prior to the workshop. However, after the workshop, only 14 trainees felt "slightly confident" with no trainee reporting feeling "not at all confident". The pre-intervention survey reinforces that internal medicine residents report low confidence in managing patients with CCOD [15]. The observed improvement in confidence suggests that the 5Ms framework may be an effective scaffold to support the integration of medical knowledge in caring for this population. These findings highlight the need for additional didactics and clinical exposures to better prepare residents for the care of this growing patient group. Interestingly, case two had only a medium level of improvement in confidence (p<0.005), which was the least medically complex patient (30-year-old with prediabetes and cough) and had the highest rated level of confidence in the pre-intervention survey. 

Although this workshop represents a novel application of the 5Ms framework, the observed increase in resident confidence mirrors the improvements in trainee self-efficacy reported in a workshop applying the framework to older adults, underscoring the high value of this shared mental model [9]. 

Strengths of the study include its execution at a large academic program with many resident participants. The workshop itself demanded minimal time commitment from busy residents. It is a workshop that can easily be adapted to already existing models of teaching (such as a traditional didactic lecture). An additional strength of this workshop is that it effectively emphasized and illustrated the importance of interprofessional teamwork and collaboration in medicine across specialties. Examples of interprofessional teamwork include working with nursing colleagues to identify and treat delirium and working with clinical pharmacists to reduce polypharmacy, and physical therapy to improve mobility. This workshop can easily be disseminated to other training programs that deal with medical complexity, with the nuances of the cases adapted to each particular specialty. Limitations of this pilot study include a single institution, dependence on self-reported subjective data, and a lack of attendance monitoring at ambulatory didactics to ensure that all residents received the information. Additional limitations include the survey attrition rate because residents' surveys were excluded if they completed only one survey (arrived late, left early, or chose not to do both surveys) and potential bias garnered by using only paired responses in the data analysis. 

As this workshop will be offered again as part of a recurring ambulatory curriculum, future iterations of the survey instruments will incorporate knowledge-based markers in addition to confidence levels, as well as spaced follow-up assessments to measure the workshop's impact. 

## Conclusions

This pilot study demonstrates that adapting the 5Ms framework beyond geriatrics is both feasible and effective for teaching residents to approach medically complex patients across the lifespan. The framework provided a shared mental model that improved residents' perceived confidence in managing complexity and reinforced the importance of interprofessional collaboration.

The findings suggest that the 5Ms can serve as a scalable and adaptable teaching tool for medical and surgical residency programs, with potential to strengthen training for populations with childhood-onset chronic diseases and other patient groups requiring complex care. Future work should build on this foundation by incorporating objective measures of knowledge and evaluating outcomes across multiple institutions.
